# Factors associated with psychiatric disorders and treatment seeking behaviour among older adults in India

**DOI:** 10.1038/s41598-021-03385-7

**Published:** 2021-12-16

**Authors:** Shobhit Srivastava, KM Sulaiman, Drishti Drishti, T. Muhammad

**Affiliations:** International Institute for Population Sciences, Mumbai, Maharashtra 400088 India

**Keywords:** Diagnosis, Geriatrics, Health services, Public health

## Abstract

Since untreated or undertreated late-life mental disorders is associated with grave consequences including poor quality of life and increased mortality rates, this study investigates the associated factors of psychiatric disorders and its treatment seeking among older adults in India. Data for this study were derived from the Longitudinal Ageing Study in India (LASI) conducted during 2017–2018. The effective sample size was 31,464 older adults aged 60 years and above. Descriptive statistics and bivariate analysis were used to present the preliminary results. Chi-square test was used to find the significance level for bivariate association. Additionally, the Heckprobit selection model was employed to fulfil the objectives. It was revealed that about 2.8% of older adults had psychiatric disorder and of those who were suffering from psychiatric disorder, 41.3% (out of 2.8%) sought medical treatment. It was found that older adults who ever worked but currently not working, who had low level of life satisfaction, had poor self-rated health, had difficulty in activities of daily living (ADL) and instrumental ADL and had symptoms of psychological distress had higher probability of suffering from psychiatric disorder in reference to their counterparts. Older adults from oldest-old age group, who were females, from poorest wealth quintile, from Scheduled Tribe and from eastern region had lower probability of seeking treatment for psychiatric disorder in reference to their counterparts. The findings of the present study urge that greater attention be devoted at detecting and preventing late-life psychiatric disorder particularly among those who are at greater risk vis., male gender, working status as “ever worked but currently not working”, having low life satisfaction, poor SRH, ADL and IADL difficulties, higher psychological distress, belonging to higher wealth quintile and rural place of residence.

## Introduction

A large proportion of people with psychiatric morbidities live in low- and middle-income countries^1,2^. Particular adverse health, social, and economic situations in those countries make geriatric population more vulnerable to develop mental illnesses^3,4^. Psychiatric disorders are shown to be leading cause of disease burden globally and a recent meta-analysis of studies in seven South Asian countries revealed that 12.2% of the population suffered from common mental disorders^5^. Similarly, the reported variable prevalence rates of mental illnesses in India ranged from 8.9 to 61.2%^6,7^. On the other hand, older adults with psychological morbidities who are endorsed with higher levels of public stigma are less likely to be engaged in seeking mental health treatment^8^. Thus, the striking underutilization of mental health services by older adults is a major public health concern world-wide and poor resource settings in particular^9^.

Demographic factors such as female sex, low education and low income status have been significantly associated with greater prevalence of mental illnesses especially among older population^10,11^. At the same time, mental disorders among older individuals improve with age and their prevalence decreases as age increases^12^. Researchers have observed that older individuals tend to be more skilled at emotional regulation compared to younger adults^13^. Moreover, studies found that greater family and social networks for care and support in terms of co-residential living and community involvement played a major role in maintaining better mental health status for older individuals^14–16^. On the other hand, several other studies found that among older adults, those with low socioeconomic status, and fewer psychological, social, and financial resources for coping with stress tend to experience higher levels of psychological distress and a low subjective wellbeing^17–19^.

However, due to several reasons including limited specialty mental health professionals who are appropriately trained in the delivery of services to older adults, age was found to be significantly associated with less likelihood of mental health service utilization in previous studies in India and other countries^20–22^. Treatment seeking is also determined by factors such as sex, social support, and several objective and subjective socioeconomic indicators^23,24^. An increased prevalence of chronic medical conditions in old age that is associated with an increase in perceived need for mental health care services has a positive impact on older adults’ chances of accessing and receiving health services for mental disorders^22,25,26^. A couple of studies suggest that factors that determine who is accessed to mental health care services such as urban place of residence, accessibility and household wealth status in India remain uncertain^27–29^.

Since untreated or undertreated late-life mental disorders is associated with numerous grave consequences, including poor quality of life and increased rates of mortality^30,31^, it is critical to understand the correlates of psychiatric disorders and explore the factors associated with seeking mental health treatment in older population. This study aims to investigate the associated factors of psychiatric disorders and its treatment seeking among older adults in India using large nationally representative survey data.

## Methods

### Data

Data for this study was utilized from the recent release of Longitudinal Ageing Study in India (LASI) wave 1^32^. The LASI is a full-scale national survey of scientific investigation of the health, economic, and social determinants and consequences of population aging in India, conducted during 2017–2018. The LASI is a nationally representative survey of over 72,000 adults aged 45 and above across all states and union territories of India^32^. The main objective of the survey is to study the health status and the social and economic well-being of older adults in India. LASI adopted a multistage stratified area probability cluster sampling design to arrive at the eventual units of observation: adults age 45 and above and their spouses irrespective of age. The survey adopted a three-stage sampling design in rural areas and a four-stage sampling design in urban areas. In each state/UT, the first stage involved the selection of Primary Sampling Units (PSUs), that is, sub-districts (Tehsils/Talukas), and the second stage involved the selection of villages in rural areas and wards in urban areas in the selected PSUs^32^. In rural areas, the households were selected from selected villages in the third stage. However, sampling in urban areas involved an additional stage. Specifically, in the third stage, one Census Enumeration Block (CEB) was randomly selected in each urban area^32^. In the fourth stage, households were selected from this CEB. The detailed methodology, with the complete information on the survey design and data collection, was published in the survey report^32^. The present study is conducted on the eligible respondents aged 60 years and above. The total sample size for the present study is 31, 464 older adults aged 60 years and above.

### Variable description

#### Outcome variables

The outcome variables were the diagnosis of neurological or psychiatric problem (s) and their treatment seeking behaviour. The diagnosis was made by any qualified health professional. The question used to assess the neurological or psychiatric problem (s) was “Which type of neurological or psychiatric problem (s) have you been diagnosed with?” the responses were 1. Depression 2. Alzheimer’s disease, Dementia 3. Psychiatric problems such as unipolar/bipolar disorder, schizophrenia etc. 4. Neurological problems such as neuropathy, convulsions, migraine, Parkinson’s etc. The variable was coded as “yes” if the respondent suffered from any of the above mentioned neurological or psychiatric problem (s) and “no” if the respondent did not suffer from any of the above mentioned neurological or psychiatric problem (s). Additionally, treatment seeking behaviour was assessed using the question “Are you currently taking any psychiatric or psychological treatment or therapy for your condition?” coded as “no” and “yes”.

#### Explanatory variables

##### Individual factors

i. Age was categorized as young old (60–69 years), old-old (70–79 years) and oldest old (80+ years).
ii. Sex was categorized as male and female.
iii. Educational status was categorized as no education/primary not completed, primary, secondary and higher.
iv. Living arrangement was categorized as living alone, living with spouse, living with children and living with others.
v. Marital status was coded as currently married, widowed and others. Others included respondents who separated/divorced/never married.
vi. Working status was categorized as currently working, ever worked but currently not working and not working.
vii. Distance from health facility was coded as “not remote i.e., less than 25 kilo-meters (kms)”, remote “25 kms or more” and “missing”. The missing values occur when the question is asked to the respondents who never went for seeking psychiatric treatment.
viii. Social participation was categorized as no and yes. Social participation was measured though the question “Are you a member of any of the organizations, religious groups, clubs, or societies”? The response was categorized as no and yes^18^.

##### Health factors

i. Life satisfaction among older adults was assessed using the questions a. In most ways my life is close to ideal; b. The conditions of my life are excellent; c. I am satisfied with my life d. So far, I have got the important things I want in life; e. If I could live my life again, I would change almost nothing. The responses were categorized as strongly disagree, somewhat disagree, slightly disagree, neither agree nor disagree, slightly agree, somewhat agree and strongly agree. Using the responses to the five statements regarding life satisfaction, a scale was constructed. The categories of the scale are ‘low satisfaction’ (score of 5–20), ‘medium satisfaction’ (score of 21–25), and ‘high satisfaction’ (score of 26–35)^32^.
ii. Self-rated health was coded as good which includes excellent, very good and good where as poor includes fair and poor^33^.
iii. Difficulty in ADL (Activities of Daily Living) was coded as no and yes. ADL is a term used to refer to normal daily self-care activities (such as movement in bed, changing position from sitting to standing, feeding, bathing, dressing, grooming, personal hygiene etc.) The ability or inability to perform ADLs is used to measure a person’s functional status, especially in the case of people with disabilities and the older adults^16,34^.
iv. Difficulty in IADL (Instrumental Activities of Daily Living) was coded as no and yes. IADLs are not necessarily related to fundamental functioning of a person, but they let an individual live independently in a community. The set of questions asked were necessary for independent functioning in the community. Respondents were asked if they were having any difficulties that were expected to last more than three months, such as preparing a hot meal, shopping for groceries, making a telephone call, taking medications, doing work around the house or garden, managing money (such as paying bills and keeping track of expenses), and getting around or finding an address in unfamiliar places^34^.
v. Psychological distress was coded as low, medium and high. Psychological distress was measured using the following questions a. How often did you have trouble concentrating? b. How often did you feel depressed? c. How often did you feel tired or low in energy? d. How often were you afraid of something? e. How often did you feel you were overall satisfied? f. How often did you feel alone? g. How often were you bothered by things that don’t usually bother you? h. How often did you feel that everything you did was an effort? i. How often did you feel hopeful about the future? j. How often did you feel happy? The response was coded as 1. Rarely or never 2. Sometimes 3. Often and 4. Most or all of the time. The response was coded as per the question in binary form 0 “Rarely or never/sometimes” and 1 “Often/ Most or all of the time” (Cronbach alpha: 0.70). A score of 0–10 was thus calculated using egen command in STATA and a variable consisting of three quintiles (low, medium and high) was made using xtile command STATA 14.

##### Household factors

i. The monthly per capita consumption expenditure (MPCE) quintile was assessed using household consumption data. Sets of 11 and 29 questions on the expenditures on food and non-food items, respectively, were used to canvas the sample households. Food expenditure was collected based on a reference period of seven days, and non-food expenditure was collected based on reference periods of 30 days and 365 days. Food and non-food expenditures have been standardized to the 30-day reference period. The MPCE is computed and used as the summary measure of consumption^32^. The variable was then divided into five quintiles i.e., from poorest to richest.
ii. Religion was coded as Hindu, Muslim, Christian, and Others.
iii. Caste was recoded as Scheduled Tribe (ST), Scheduled Caste (SC), Other Backward Class (OBC), and Others.
iv. Place of residence was categorized as rural and urban.
v. The region was coded as North, Central, East, Northeast, West, and South.

### Statistical approach

Descriptive statistics and bivariate analysis were used to present the preliminary results. Chi-square test was used to find the significance level for bivariate association. Additionally, the study employed the heckprobit selection model, which is a two-equation model. First, there is a selection model (in this study, referring to “Have you been diagnosed with neurological or psychiatric problem (s)? (Yes or No)”). Secondly, there is an outcome model with a binary outcome (in this study refers to “Are you currently taking any psychiatric or psychological treatment or therapy for your condition? (Yes or No)”). The model provides a two-step analysis and deals with the zero-sample issue, based on which it can accommodate the heterogeneity (i.e., shared unobserved factors) between older adults and then address the endogeneity (between diagnosed with neurological or psychiatric problem (s) and its treatment seeking behaviour) for older adults in India.

The Heckman model is identified when the same independent variables in the selection equation appear in the outcome equation. However, this does not provide precise estimates in the outcome equation because of high multicollinearity; it was suggested to have at least one independent variable that appears in the selection equation and not in the outcome equation. A p-value of less than 0.05 was considered statistically significant. The study used heckprobit model because it is a two-step model and provides the reliable estimates when the outcome variables are sequential in nature. In the present study the factors for neurological or psychiatric problem (s) are estimated and then sequentially, factors for its treatment seeking behaviour are analysed.

The probit model with sample selection assumes that there exists an underlying relationship:

latent equation $${y}_{j}={x}_{j}\beta +{u}_{1j},$$

such that we observe only the binary outcome

probit equation $${y}_{i}^{probit}=\left({y}_{j}>0\right).$$

The dependent variable, however, is not always observed. Rather, the dependent variable for observation j is observed if:

selection equation $${y}_{i}^{select}= ({z}_{j}\gamma +{u}_{2j}>0,$$

where, $${u}_{1}\sim N (0, 1)$$, $${u}_{2}\sim N (0, 1)$$, $$Corr \left({u}_{1}, {u}_{2}\right)=\rho$$.

When $\rho$≠ 0, standard probit techniques applied to the first equation yield biased results.

Heckprobit provides consistent, asymptotically efficient estimates for all the parameters in such models. For the model to be well identified, the selection equation should have at least one variable that is not in the probit equation. Otherwise, the model is identified only by functional form, and the coefficients have no structural interpretation.

### Ethics approval and consent to participate

The data are freely available in the public domain and survey agencies that conducted the field survey for the data collection have collected prior informed consent from the respondents. The Indian Council of Medical Research (ICMR) and all partner institutions extended the necessary guidance and ethical approval for conducting the LASI survey.

## Results

### Socio-economic and health profile of older adults in LASI

Table 1 represents the socio-economic profile of the older adults in India. The table reveals that 59% of the older adults were among the young old age group while only 11% of the older adults were among the oldest old age group. More than half of the older adults were female. About 68% of the older adults had education status as “No education/Primary education not completed”. Around 68% of the older adults reported that they were living with their children while only 5.7% of the older adults were living alone. Nearly 62% of the older adults had marital status as “currently married” while 36% of the older adults had marital status as “widowed”. About 43% of the older adults ever worked but currently not working among the working status category while 31% of the older adults were currently working. About 7.8% of older adults had a remote location in terms of distance from health facility. Nearly, 96% of the older adults reported to have no social participation while only 4.5% of the older adults reported to have social participation. Around 44% and 35% of the older adults reported to have high level and low level of satisfaction in life respectively. Over 47% of the older adults had poor self-rated health. About 24% and 49% of the older adults reported to have difficulty in ADL and IADL respectively. Nearly 39% of the older adults had low psychological distress while 29% of the older adults had high psychological distress**.**

**Table 1:** Socio-economic profile of older adults in India, 2017–2018.

Background factors	Sample	Percentage
**Individual factors**		
Age group		
Young-old	18,410	58.5
Old-old	9501	30.2
Oldest-old	3553	11.3
Sex		
Male	14,931	47.5
Female	16,533	52.6
Education status		
No education/primary not completed	21,381	68.0
Primary completed	3520	11.2
Secondary completed	4371	13.9
Higher and above	2191	7.0
Living arrangements		
Alone	1787	5.7
With spouse	6397	20.3
With children	21,475	68.3
Others	1805	5.7
Marital status		
Currently married	19,391	61.6
Widowed	11,389	36.2
Others	684	2.2
Working status		
Working	9680	30.8
Ever worked but currently not working	13,470	42.8
Not working	8314	26.4
Distance from health facility		
Not remote	15,408	49.0
Remote	2469	7.8
Missing	13,588	43.2
Social participation		
No	30,053	95.5
Yes	1411	4.5
Life satisfaction		
Low	10,974	34.9
Medium	6754	21.5
High	13,737	43.7
**Health factors**		
Self-rated health		
Good	16,582	52.7
Poor	14,882	47.3
Difficulty in ADL		
No	23,802	75.7
Yes	7662	24.4
Difficulty in IADL		
No	16,130	51.3
Yes	15,334	48.7
Psychological distress		
Low	12,135	38.6
Medium	10,216	32.5
High	9114	29.0
**Household factors**		
MPCE index		
Poorest	8803	28.0
Poorer	5806	18.5
Middle	5681	18.1
Richer	5556	17.7
Richest	5618	17.9
Religion		
Hindu	25,871	82.2
Muslim	3548	11.3
Christian	900	2.9
Others	1145	3.6
Caste		
Scheduled Caste	5949	18.9
Scheduled Tribe	2556	8.1
Other Backward Class	14,231	45.2
Others	8729	27.7
Place of residence		
Rural	22,196	70.6
Urban	9268	29.5
Region		
North	3960	12.6
Central	6593	21.0
East	7439	23.6
Northeast	935	3.0
West	5401	17.2
South	7136	22.7
Total	31,464	100.0

*ADL* Activities of daily living, *IADL* Instrumental activities of daily living, *MPCE* Monthly per capita consumption expenditure.

**Figure 1:**
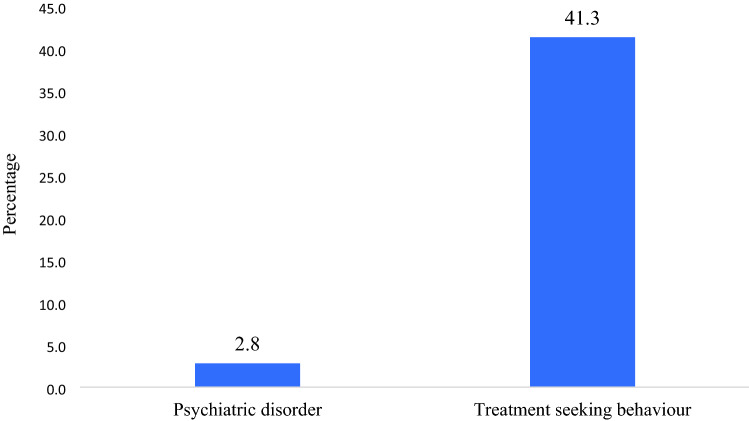
Percentage of psychiatric disorder and treatment seeking behaviour among older adults.

Figure 1 revealed that about 2.8% of older adults had psychiatric disorder and of the total older adults who were suffering from psychiatric disorder, 41.3% (out of 2.8%) went to seek treatment.

### Percentage of older adults who reported psychiatric disorder and their treatment seeking behaviour

Table 2 depicts Percentage of older adults who reported psychiatric disorder and their treatment seeking behaviour in India. It was found that the percentage of psychiatric disorder was highest among oldest old age group (4.5%). Higher percentage of older adults who were from other marital status category i.e. separated/never married/divorced had psychiatric disorder (4.4%). The share of psychiatric disorder was highest among the older adults who were currently not working (3.3%). The percentage of psychiatric disorder was higher among the older adults who had no social participation (2.9%). Higher percentage of older adults who reported to have low level of satisfaction in life was suffering from psychiatric disorder (3.8%). The share of psychiatric disorder was higher among the older adults who reported to have poor self-rated health (4.0%). The percentage of psychiatric disorder was higher among the older adults who had difficulty in ADL (5.9%) and AIDL (4.3%) respectively. The share of older adults suffering from psychiatric disorder was directly proportional to the level of the psychological distress. The share of psychiatric disorder among the caste groups was highest among the older adults from the Scheduled Caste (3.5%). The prevalence of psychiatric disorder was higher among the older adults who had rural place of residence (2.9%). Among the regions, the share of older adults who had psychiatric disorder was highest in southern region (4.1%).

**Table 2:** Percentage of older adults reported psychiatric disorder and their treatment seeking behaviour in India, 2017–2018.

Background factors	Psychiatric disorder		Treatment seeking	
	Percentage	p-value	Percentage	p-value
**Individual factors**				
Age group		0.001		0.007
Young-old	2.4		47.9	
Old-old	2.9		39.4	
Oldest-old	4.5		26.3	
Sex		0.944		0.045
Male	2.7		49.0	
Female	2.9		34.9	
Education status		0.758		0.001
No education/primary not completed	2.9		38.2	
Primary completed	2.8		44.5	
Secondary completed	2.5		50.8	
Higher and above	2.5		53.2	
Living arrangements		0.145		0.558
Alone	3.2		30.7	
With spouse	2.8		49.8	
With children	2.7		40.0	
Others	3.6		40.0	
Marital status		0.001		0.016
Currently married	2.6		46.3	
Widowed	3.1		32.9	
Others	4.4		58.8	
Working status		0.001		0.229
Working	1.9		51.7	
Ever worked but currently not working	3.2		42.5	
Not working	3.3		32.9	
Distance from health facility				0.001
Not remote	–		38.6	
Remote	–		73.1	
Missing	–		35.1	
Social participation		0.03		
No	2.9			
Yes	1.7			
Life satisfaction		0.001		
Low	3.8			
Medium	3.0			
High	1.9			
**Health factors**				
Self-rated health		0.001		
Good	1.7			
Poor	4.0			
Difficulty in ADL		0.001		
No	1.9			
Yes	5.9			
Difficulty in IADL		0.001		
No	1.4			
Yes	4.3			
Psychological distress		0.001		
Low	2.0			
Medium	2.3			
High	4.4			
**Household factors**				
MPCE index		0.047		0.001
Poorest	2.4		28.1	
Poorer	3.5		35.7	
Middle	3.1		45.4	
Richer	2.9		48.5	
Richest	2.5		55.6	
Religion		0.25		0.109
Hindu	2.8		39.4	
Muslim	3.0		56.1	
Christian	3.4		34.4	
Others	1.8		47.9	
Caste		0.001		0.001
Scheduled Caste	3.5		31.3	
Scheduled Tribe	2.1		38.9	
Other Backward Class	2.7		43.7	
Others	2.7		47.0	
Place of residence		0.001		0.003
Rural	2.9		36.4	
Urban	2.7		54.3	
Region		0.001		0.1
North	1.9		54.4	
Central	2.0		46.0	
East	3.6		28.7	
Northeast	2.2		61.6	
West	1.7		44.2	
South	4.1		45.1	

*ADL* Activities of daily living, *IADL* Instrumental activities of daily living, p-value is based on chi square test, *MPCE* Monthly per capita consumption expenditure.

The Percentage of older adults seeking treatment for psychiatric disorder was higher among the young-old age group (47.9%). The male older adults seeking treatment for psychiatric disorders was higher as compared to the female older adults (49% vs 35%). Higher percentage of older adults who had higher and above educational status was seeking treatment for psychiatric disorder (53.2%). The percentage of older adults seeking treatment for psychiatric disorder was higher among separated/never married/divorced (58.8%). A higher percentage of older adults living in remote locations from the health facility in comparison to not remote counterparts was seeking treatment for psychiatric disorder (73.1% vs 38.6%). Higher percentage of older adults from richest wealth index were seeking treatment for psychiatric disorder (55.6%). The percentage of older adults seeking treatment for psychiatric disorder was higher among other caste category (47.0%). Higher percentage of older adults from urban place of residence was seeking treatment for psychiatric disorder (54.3%).

### Heckprobit model estimates for psychiatric disorder and treatment seeking behaviour among older adults

Table 3 represents the heckprobit model estimates for psychiatric disorder and treatment seeking behavior among older adults in India. It was found that the female older adults were 0.08 times less likely to suffer from psychiatric disorder in reference to the male older adults [Coef: − 0.08; confidence interval (CI) − 0.17, 0.01]. The older adults who ever worked but currently not working were 0.10 times significantly more likely to suffer from psychiatric disorder in comparison to the older adults who were currently working [Coef: 0.10; CI 0.01, 0.19]. The older adults who reported to have low level of satisfaction in life were 0.07 times more likely to suffer from psychiatric disorder in reference to the older adults who reported to have high level of satisfaction in life [Coef: 0.07; CI − 0.01, 0.15]. The probability of older adults who had poor self-rated health were 0.29 times significantly more likely to suffer from psychiatric disorder in comparison of the older adults who had good self-rated health [Coef: 0.29; CI 0.22, 0.36]. The chances of older adults who had difficulty in ADL and IADL were 0.20 times [Coef: 0.20; CI 0.12, 0.28] and 0.27 times [Coef: 0.27; CI 0.19, 0.35] significantly more likely to suffer from psychiatric disorder respectively in comparison of the older adults who had no difficulty in ADL and IADL. The older adults who had high level of psychological distress were 0.30 times significantly more likely to suffer from psychiatric disorder in reference to the older adults who had low level of psychological distress [Coef: 0.30; CI 0.21, 0.38].The probability of psychiatric disorder was lower among the older adults who belonged to the poorest wealth index in comparison of the older adults who belonged to the richest wealth index [Coef: − 0.13; CI − 0.26, 0.01].The older adults from other religious category were 0.16 times significantly more likely to suffer from psychiatric disorder in reference to the older adults from Hindu religion [Coef: 0.16; CI 0.02, 0.31]. The older adults from rural place of residence were 0.09 times significantly less likely to suffer from psychiatric disorder in reference to the older adults who were from urban place of residence [Coef: − 0.09; CI − 0.17, − 0.01]. The probability of older adults suffering from psychiatric disorder was highest among the south region in reference to the older adults from north region [Coef: 0.32; CI 0.21, 0.43].

**Table 3:** Heckprobit model estimates for psychiatric disorder and treatment seeking behaviour among older adults in India, 2017–2018.

Background factors	Outcome equation	Selection equation
	Psychiatric disorder	Treatment seeking
	Coef.	Coef.
**Individual factors**		
Age group		
Young-old	Ref.	Ref.
Old-old	− 0.05 (− 0.12, 0.03)	− 0.11 (− 0.33, 0.11)
Oldest-old	0.03 (− 0.07, 0.14)	− 0.33** (− 0.63, − 0.02)
Sex		
Male	Ref.	Ref.
Female	− 0.08* (− 0.17, 0.01)	− 0.22* (− 0.47, 0.03)
Education status		
No education/primary not completed	− 0.11 (− 0.25, 0.03)	− 0.01 (− 0.39, 0.37)
Primary completed	− 0.12 (− 0.27, 0.04)	0.01 (− 0.43, 0.43)
Secondary completed	− 0.05 (− 0.19, 0.09)	0.10 (− 0.30, 0.50)
Higher and above	Ref.	Ref.
Living arrangements		
Alone	0.01 (− 0.20, 0.20)	− 0.10 (− 0.67, 0.48)
With spouse	0.05 (− 0.12, 0.23)	− 0.35 (− 0.86, 0.16)
With children	0.02 (− 0.14, 0.17)	− 0.57* (− 1.02, − 0.12)
Others	Ref.	Ref.
Marital status		
Currently married	Ref.	Ref
Widowed	− 0.06 (− 0.15, 0.03)	− 0.09 (− 0.36, 0.17)
Others	0.11 (− 0.09, 0.31)	− 0.43 (− 1.00, 0.13)
Working status		
Working	Ref.	Ref.
Ever worked but currently not working	0.10** (0.01, 0.19)	− 0.18 (− 0.44, 0.08)
Not working	0.08 (− 0.02, 0.19)	− 0.01 (− 0.33, 0.32)
Distance from health facility		
Not remote	–	Ref.
Remote	–	0.82* (0.53, 1.11)
Missing	–	0.07 (− 0.25, 0.39)
Social participation		
No	0.04 (− 0.10, 0.19)	
Yes	Ref.	
Life satisfaction		
Low	0.07* (− 0.01, 0.15)	
Medium	0.06 (− 0.02, 0.14)	
High	Ref.	
**Health factors**		
Self-rated health		
Good	Ref.	
Poor	0.29*** (0.22, 0.36)	
Difficulty in ADL		
No	Ref.	
Yes	0.20*** (0.12, 0.28)	
Difficulty in IADL		
No	Ref.	
Yes	0.27*** (0.19, 0.35)	
Psychological distress		
Low	Ref.	
Medium	0.16*** (0.07, 0.24)	
High	0.30*** (0.21, 0.38)	
**Household factors**		
MPCE index		
Poorest	− 0.13* (− 0.26, 0.01)	− 0.71*** (− 1.11, − 0.30)
Poorer	− 0.05 (− 0.17, 0.07)	− 0.34* (− 0.69, 0.01)
Middle	0.03 (− 0.11, 0.11)	− 0.24 (− 0.55, 0.07)
Richer	0.01 (− 0.09, 0.11)	− 0.02 (− 0.31, 0.26)
Richest	Ref	Ref
Religion		
Hindu	Ref.	Ref.
Muslim	− 0.02 (− 0.12, 0.08)	0.17 (− 0.12, 0.47)
Christian	− 0.04 (− 0.18, 0.09)	− 0.17* (− 0.57, 0.24)
Others	0.16*** (0.02, 0.31)	− 0.38 (− 0.87, 0.10)
Caste		
Scheduled Caste	− 0.01 (− 0.12, 0.1)	− 0.25 (− 0.57, 0.07)
Scheduled Tribe	− 0.03 (− 0.15, 0.1)	− 0.39** (− 0.78, − 0.13)
Other Backward Class	0.03 (− 0.06, 0.11)	− 0.21 (− 0.47, 0.06)
Others	Ref.	Ref.
Place of residence		
Rural	− 0.09** (− 0.17, − 0.01)	0.06 (− 0.16, 0.29)
Urban	Ref.	Ref.
Region		
North	Ref.	Ref.
Central	0.10 (− 0.03, 0.24)	− 0.12 (− 0.55, 0.31)
East	0.11* (− 0.01, 0.23)	− 0.37** (− 0.75, − 0.11)
Northeast	0.08 (− 0.08, 0.23)	− 0.01 (− 0.50, 0.48)
West	0.14*** (0.01, 0.27)	− 0.01 (− 0.42, 0.40)
South	0.32*** (0.21, 0.43)	− 0.22 (− 0.59, 0.16)

*Ref* Reference, *CI* Confidence interval, *ADL* Activities of daily living; *IADL* Instrumental activities of daily living; *MPCE* Monthly per capita consumption expenditure.

*if *p* < 0.10, **if *p* < 0.05 and ***if *p* < 0.01.

The older adults from oldest-old age group were 0.33 times significantly less likely to seek treatment for psychiatric disorder in comparison of the older adults from young-old age group [Coef: − 0.33; CI − 0.63, − 0.02]. The probability of female older adults was 0.22 times less likely to seek treatment for psychiatric disorder in reference to the male older adults [Coef: − 0.22; CI − 0.47, 0.03]. The older adults who lived with their children were 0.57 times less likely to seek treatment for psychiatric disorder in reference to the older adults who had living arrangement as “others” [Coef: − 0.57; CI − 1.02, − 0.12]. The chances of older adults who lived in remote areas from the health facility were 0.82 times significantly less likely to seek treatment for psychiatric disorder in comparison to their counterparts [Coef: 0.82; CI: 0.53, 1.11]. The chances of older adults from poorest wealth index were 0.71 times significantly less likely to seek treatment for psychiatric disorder in comparison of the older adults from richest wealth index. [Coef: − 0.71; CI − 1.11, 0.30]. The older adults from Christian religious category were 0.17 times less likely to seek treatment for psychiatric disorder in comparison to the older adults from Hindu religion [Coef: − 0.17; CI − 0.57, 0.24]. The older adults from Scheduled Tribes were 0.39 times were significantly less likely to seek treatment for psychiatric disorder in comparison of older adults from others caste category [Coef: − 0.39; CI − 0.78, − 0.13]. The probability of older adults from eastern region were 0.37 times significantly less likely to seek treatment for psychiatric disorder in reference to the older adults from northern region [Coef: − 0.37; CI − 0.75, − 0.11].

## Discussion

The present study of the determinants of psychiatric disorder and its treatment seeking using a country representative survey data shows that 2.8 percent of older adults suffered from psychiatric disorder and 41.3% of them chose to seek treatment. Oldest-old participants have the highest prevalence of the psychiatric disorder, and is consistent with previous researches that showed the increased prevalence of the psychiatric disorder among older adults by increasing age^35,36^.

The multivariable analysis in the present study showed the substantial gender difference in the prevalence of psychiatric disorder; where older men were more likely to develop psychiatric disorders than older women. This is in contrary with earlier studies showing that older women are more likely to experience psychiatric disorders compared to older men^37–39^. This might be attributed to the fact that the gender differences in mental disorders appear to be narrowed by increasing age^40^, which is evident in bivariate results with slight gender difference. Also, the lower prevalence of psychiatric disorder in our study might have led to lack of power or type-2 error which results in underestimation of the particular association. Furthermore, the study showed that older adults who are actively working are less likely to suffer from psychiatric disorder than those who ever worked but currently not working or retired; earlier studies also point out that being unemployed or retired has a devastating impact on mental wellbeing in old age^41,42^. Previous research also suggests that lack of proper financial savings and assets are the primary reasons that older adults prefer to stay in their occupation^43,44^. In addition, with regard to economic status, the current analysis revealed that older adults from the poorest wealth quintile were less likely to suffer from psychiatric disorder than the richest older adults. Another important finding of our study is that older individuals living in urban areas are more likely to suffer from psychiatric disorders than their rural counterparts. Still, some studies point out that the rural resident older adults are at increased risk of psychiatric disorders than urban older adults^45^. However, this can vary by the context and the regions. In developing countries, this association was not strongly observed^46^; further research should concentrate on covering this variation.

The high life satisfaction and good SRH reduce psychiatric disorder among older adults^47,48^. In accordance with this, according to our study, people with low life satisfaction and poor SRH were more likely to suffer from psychiatric disorders. In a broader sense, fruitful ageing covers components such as life satisfaction, physical health, and quality of life^49^. When it comes to functional health, older adults with difficulty in ADL and IADL had higher risk of suffering from psychiatric disorder and is similar to findings of earlier studies^50,51^. It is also evident from the research that older adults who have higher psychological distress are substantially more likely to suffer from a psychiatric disorder indicating that as evidence suggests, the distress symptoms should be considered as screening for recognising psychiatric morbidity among older population^52^.

Several studies show that healthcare seeking or seeking health treatment reduces by increasing age^53,54^; in this study seeking treatment for psychiatric disorder was found to be significantly lower among oldest-old group than young-old adults. This underscores the stigma that is related to having a mental disorder in old age that has adverse impacts on seeking mental health services^8^. The analysis showed that older women are significantly less likely to seek treatment for a psychiatric disorder than older men; this corresponds to a recent study^55^, pointing out the gender differential in healthcare utilisation in India among older adults. Similarly, according to this study, living arrangements are related to treatment-seeking for psychiatric disorder of older adults. This result is in line with several studies that suggested the impact of living arrangement on the healthcare seeking of older adults in India^56–58^. The analyses reveal that older adults who live alone are less likely to seek treatment for their psychiatric illnesses than older adults in other living arrangements. The household economic status has a profound implication on the health and health-seeking behaviour of older people^58–61^; and poor financial conditions act as a significant determinant of lack of treatment-seeking, whereas, the illness is considered a part of ageing and treatment seems a waste of money^62^. In this study, even after adjusting for health service accessibility, older adults from the poorest economic background were less likely to seek treatment for psychiatric disorder than those from the richest households. The increased odds of seeking treatment among older adults residing remote from health services may be related to the higher cost and affordability of the services. This aspect also needs to be further investigated.

It is essential to study the health-seeking behaviour among people with psychiatric symptoms comprehensively and attention must be paid for older population since they are more susceptible to adverse consequences of lack of treatment^63^. To best of our knowledge, this is the first study addressing the factors associated with psychiatric disorders and treatment-seeking behaviour among older adults in India. The strength of this study is that the data provide exhaustive information of older population which were obtained from a comprehensive, nationally-representative sample of older people aged 60 and above, improving external validity and generalisation. However, the current study has several limitations to be acknowledged. Notably, the study was cross-sectional; therefore, any causal pathways cannot be definitively determined from the findings. The forthcoming followed-up wave 2 of the LASI data may help understand the magnitude of psychiatric disorder and different types of treatment-seeking among aging population. Again, the psychiatric disorder was self-reported and was measured by considering any one of the neurological or psychiatric problems out of the listed questions used to assess the neurological or psychiatric problem in the survey. Future studies should address the impact of other potential risk factors for particular mental illnesses such as depression, Alzheimer’s disease, dementia, Parkinson’s disease, etc. and the differential treatment-seeking for those illnesses among older Indian adults.

## Conclusion

The findings of the present study urge that greater attention be devoted at detecting and preventing late-life psychiatric disorder particularly among those who are at greater risk vis., male gender, working status as “ever worked but currently not working”, having low life satisfaction, poor SRH, ADL and IADL difficulties, higher psychological distress, belonging to higher wealth quintile and rural place of residence. Similarly, results suggest that factors such as oldest age group, female gender, co-residential living, poorest wealth quintile and lower caste groups are at increased risk for not seeking mental treatment among older adults. Thus, the treatment for psychiatric disorders in primary-care units must be availed to detect and control the disorder prevalence through modifying some of the risk factors and promote the mental health treatment seeking among older individuals especially those from poor socioeconomic backgrounds. Additional research on geriatric mental health services and efforts that clearly define and distinguish psychiatric disorder in late life as a treatable social problem are needed to elevate the priority of seeking treatment for age-related mental illnesses.

## Data Availability

The study utilizes a secondary source of data that is freely available in the public domain through a request from https://iipsindia.ac.in/sites/default/files/LASI_DataRequestForm_0.pdf.
